# Patterns Prediction of Chemotherapy Sensitivity in Cancer Cell lines Using FTIR Spectrum, Neural Network and Principal Components Analysis

**Published:** 2012

**Authors:** Rezvan Zendehdel, Ali Masoudi-Nejad, Farshad H. Shirazi

**Affiliations:** a *Student Research Committee, Shahid Beheshti University of Medical Sciences, Tehran, Iran. *; b *Laboratory of Systems Biology and Bioinformatics (LBB), Institute of Biochemistry and Biophysics and COE in Biomathematics, University of Tehran, Tehran, Iran. *; c *Pharmaceutical Research Sciences Center, Shahid Beheshti University of Medical Sciences, Tehran, Iran. *; d *Department of Toxicology and Pharmacology, School of Pharmacy, Shahid Beheshti University of Medical Sciences, Tehran, Iran.*

**Keywords:** Drug resistant, Fourier transform infrared, Principle component analysis, Artificial neuronal network, Linear discriminate analysis, Pattern recognition

## Abstract

Drug resistance enables cancer cells to break away from cytotoxic effect of anticancer drugs. Identification of resistant phenotype is very important because it can lead to effective treatment plan. There is an interest in developing classifying models of resistance phenotype based on the multivariate data. We have investigated a vibrational spectroscopic approach in order to characterize a sensitive human ovarian cell line, A2780, and its cisplatin-resistant derivative, A2780-cp. In this study FTIR method have been evaluated via the use of principal components analysis (PCA), ANN (artificial neuronal network) and LDA (linear discriminate analysis). FTIR spectroscopy on these cells in the range of 400-4000 cm^-1^ showed alteration in the secondary structure of proteins and a CH stretching vibration. We have found that the ANN models correctly classified more than 95% of the cell lines, while the LDA models with the same data sets could classify 85% of cases. In the process of different ranges of spectra, the best classification of data set in the range of 1000-2000 cm^-1^ was done using ANN model, while the data set between 2500-3000 cm^-1^ was more correctly classified with the LDA model. PCA of the spectral data also provide a good separation for representing the variety of cell line spectra. Our work supports the promise of ANN analysis of FTIR spectrum as a supervised powerful approach and PCA as unsupervised modeling for the development of automated methods to determine the resistant phenotype of cancer classification.

## Introduction

Ovarian cancer is the seventh most frequent cancer in women worldwide and it accounts for 5% of all cancers in women (1). Cisplatin is a commonly used chemotherapeutic agent that used for treatment of many types including testicular, ovarian, cervical, head and neck, non-small cell lung and lymphoma (2, 3). Resistance to cisplatin therapy is a serious hurdle for successful treatment plan. That is why identification of resistant cells at the beginning of a patient treatment saves time and is very critical for the therapy outcome**.** There is an increasing interest in the use of molecular data from human resistant neoplasm for chemotherapy purposes (4, 5). To our knowledge**,** it is not yet clear how to use molecular data to acquire diagnostic information. Therefore cancer chemotherapy investigators have been looking for a suitable, simple and fast sensitivity prediction method for long time.

Optical spectroscopy techniques, such as fluorescence, Raman, and infrared, which are sensitive to biochemical composition of samples, have shown potentials to discriminate tissues (6-8). There is an increasing interest for using FTIR to a large number of different applications. This method has been used to investigate the biochemical composition of cells (9), as well as the study of normal and malignant tissues (10, 11). These literatures have shown that the FTIR technique can be used to detect a cell phenotype or an illness with a good level of sensitivity. Various algorithms have been developed to accurately classify tumor cells. Many studies during the past five decades have used multivariate analysis of the data (12-15). Most of these methods have led to the development of analytical instruments that are currently approved by the Food and Drug Administration for the routine screening of gynecologic smears from the same organ (16, 17). Two different methods of supervised and unsupervised have been used in classification techniques. “Unsupervised” technique such as PCA (principle component analysis) attempts to detect relationships of data without any information about the classification of the data. This method tracks those data in different groups that are correlated with each other. In contrast “supervised” methods such as linear discriminate. 

analysis (LDA) and artificial neural networks (ANN) are trained using data that is labeled with the correct answer (14). 

In this study, we attempt to apply three different methods of PCA, LDA and ANN in the discrimination of FTIR spectroscopic results from sensitive human ovarian cell line, A2780, and its resistant derivative, A2780-CP. We are presenting the success of mixed classification methods in the characterization of differences between the spectra of resistant and sensitive phenotypes.

## Experimental

*Cell lines*

A2780 (human ovarian carcinoma-sensitive to cisplatin) and A2780-CP (human ovarian carcinoma-resistant to cisplatin) cell lines were obtained from Pasture Institute National Cell Bank of Iran (Tehran, Iran). All cell lines were grown in RPMI-1640 medium and supplemented with 10% heat inactivated fetal bovine serum, antibiotics: penicillin, streptomycin (all chemicals from Sigma). Cells were maintained at 37 °C in humidified atmosphere containing 5% CO_2_.

*Cell preparation for spectroscopy*

The following procedure was similarly applied for both sensitive and resistant cell lines. Cells were trypsinized from the original flask and seeded in 25 cm^2^ flasks with fresh medium to reach the logarithmic phase of growth curve. After that, cells were washed twice in saline (0.9% NaCl), suspend and centrifuged at 1000 rpm for 5 min, then resuspended in saline to obtain a concentration of 1 × 10^5^ cells. 10 μL of each cell suspension was placed on a zinc selenide sample carrier which was dehydrated in a vacuum cabin (0.8 bar) for approximately 4 min. These plates were then used for FTIR spectroscopy.

**Figure 1:**
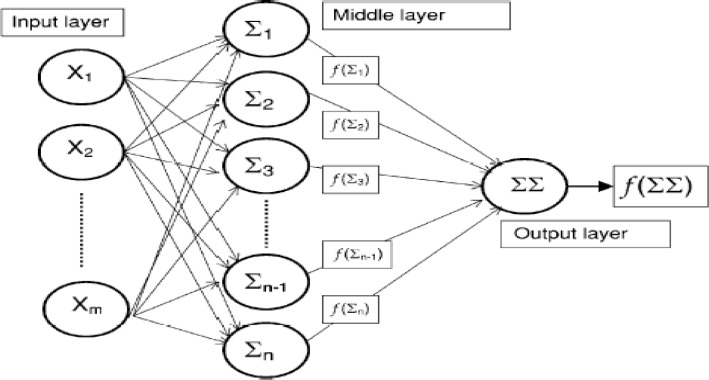
Artificial neural network (ANN) architecture (31)

*FTIR spectroscopy*

Thin dried films of cell suspensions on the Zinc selenide window were used for FTIR spectroscopy using a WQF-510 (Rayleigh Optics, China) spectrometer, equipped with a KBr beam splitter and a DLaTGS (deuterated Lantanide triglycine sulphate) detector. The whole system was continuously purged with N2 (99.999%). In each spectrum, 100 scans were collected at a resolution of 4 cm^-1 ^for every wave number between 400 and 4000 cm^-1^. These experimental conditions for each cell line and situation were kept constant for all the measurements. Each single spectrum was baseline corrected and then normalized in order to have the range spanning from 0 to 1.

*Data analysis*

A total of 60 FTIR spectrums between 1000-3000 cm^-1^ have been used in this study as the dataset. Distribution of different FTIR spectra was equal for types of A2780 and A2780-CP cell lines.

*Linear discriminate analysis (LDA)*

The basic theory of LDA is to classify the dependent variable by dividing an n-dimensional feature space into two regions that are separated by a hyper plane which is denoted by a linear discriminate function. In LDA, observations from each case are compared with others to provide models for groups of data (18). This procedure generates a discriminate function based on linear combinations of the predictor variables that provide the best discrimination among the groups. The LDA can be expressed as:

D = *β*_0_ + *β*_1_ X_1_ + *β*_2_ X_2_ +· · ·+ *β*_n_ X_n,(1)_

where D represents the discriminate score, *β*_0_ is the intercept term, and *β*_n_ (i=1, . . ., n) represents the *β* coefficient associated with the corresponding explanatory variable Xi (i=1, . . ., n) (18).

The data were analyzed by multivariate (LDA) linear discriminate analysis tool of MATLAB software using cell types as the dependent variable and the absorbance of FTIR spectra as independent variables. The same data sets used for ANN were used for the LDA analysis.

*Artificial neural networks analysis*

Artificial neural networks (ANN) are computerized mathematical models designed to mimic the architecture of the brain. They are able to detect nonlinearity, making them capable of learning and adaptability (19). Neurons are the main processing units in this model and might contain the initial data or resulted data from the previous layers of neurons. Neurons are organized in parallel layers: input, hidden (single or multiple), and output (Figure 1). Neurons process the data using a variety of mathematical functions (15). Multiple layer perceptron neuronal networks were designed using MATLAB. The output of FTIR spectrum as absorbance percent in different wave number have been used for input layer. The output layer consisted of two output neurons, one to classify the A2780 category and the others for A2780-CP data.

*Principle component analysis*

PCA is a well-known method of dimension reduction. The basic idea of PCA is to reduce the dimensionality of a data set, while retaining as much as possible the variation present in the original predictor variables. In mathematical terms, PCA sequentially maximizes the variance of a linear combination of the original predictor variables (20). The same data sets used for ANN and LDA were used for the PCA analysis.

## Results and Discussion

*Spectrum alteration*

**Figure 2:**
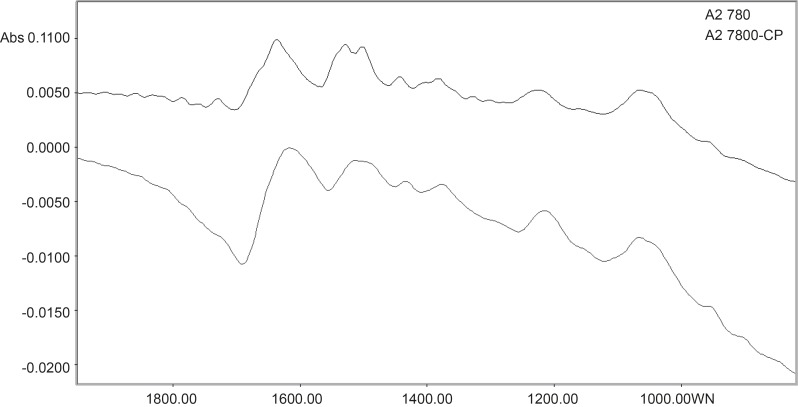
Spectral features of A2780 and A2780-CP cell lines in the range of 1900-700 cm^-1^

Spectral features of A2780 and A2780-CP cell lines are shown for the range of 1900-700 cm^-1 ^in Figure 2. The normalized FTIR spectra in this region showed alterations in different spectrum areas. Comparison between spectra showed at least three areas of variation: 

There is a peak about 1636 cm^-1^ which can be related to *β*-sheet secondary structure of amid I (21). In the ovarian human resistant cell the peak of about 1636 cm^-1^ shifted toward the lower wave numbers. Moreover there is a positive shoulder peak at 1672 cm^-1^ in the sensitive cell line but not in the resistant cell line. The band at 1672 cm^-1^ is assigned to turn in the secondary structure of amid I (22). In A2780-cp cell the band of *β*-sheet are broader than sensitive cell, this might be related to the conversion of some amid I proteins with turn secondary structure to *β*-sheet structure conformation in resistant ovarian cell.

The vibration band at 1530 cm^-1^ is assigned to *β*-sheet secondary structure of amid II (22). In resistant cell, there is a broadband at 1530 cm^-1^ while there are two bands at 1530 and 1540 cm^-1^ in the sensitive cell line. The band at 1540 cm^-1^ is assigned to *α*-secondary structure of amid II (22). This might reflect of a clear margin of *α*-secondary and *β*-sheet structure of amid II in sensitive cell. In the resistant cell, on the other hand, a possible conversion of one of these structures to other has resulted in a broadband at 1530 cm^-1^. 

The vibration bands at 1380 cm^-1^ is assigned to glycoprotein (23). There is a peak at this band in the A2780 cell line spectra which is shifted to 1374 cm^-1^ in the A2780-CP cell lines.

 It was hypothesized that protein conformational changes might be related to resistant. Moreover glycoprotein molecules of resistant cells have weaker chemical interaction than sensitive cell. This shows more free glycoprotein site in resistant cell than sensitive type. Expression of Pgp in resistant ovarian cancer cell lines (24) could be influenced this event. 

**Figure 3:**
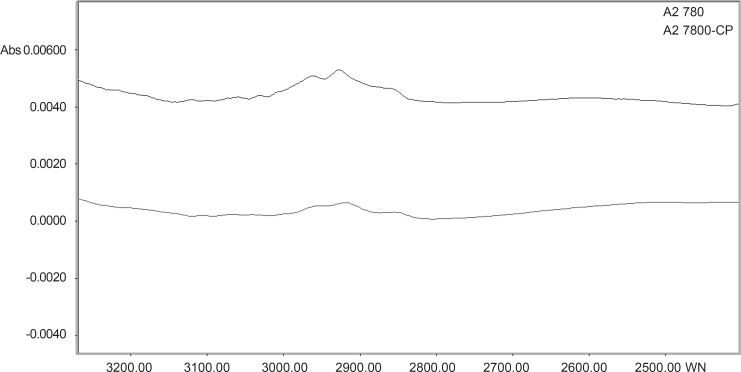
The normalized FTIR Spectral features of A2780 and A27-80-CP cell lines in the range of 3300-2700 cm^-1^

The normalized FTIR spectra of A2780, A2780-CP cell lines in region 3300-2700 cm^-1^ are shown in Figure 3. The CH stretching region (3000-2800 cm^-1^) contains the asymmetric and symmetric membrane lipids. CH_2_ symmetric and asymmetric stretching vibration bands are appeared at 2920 and 2852 cm^-1 ^(21). CH_2_ stretching vibration band shifted to higher wave number in sensitive cell line. In sensitive cell line, on the other hand, the intensity of CH_2_ stretching (at 2920 and 2852 cm^-1^) and CH_3_ stretching vibrations (at 2950 cm^-1^) are higher than resistant cell line. Our research is representing alterations in the lipophilicity of cell membrane between resistant and sensitive cells.

*Data processing*

The FTIR data of A2780 and A2780-cp cell lines were sorted randomly into 20 different data sets (numbered 1 to 20) each composed of 44 training variables and 16 testing variable. Models 1 to 5 used all FTIR wave number from 1000-3000 cm^-1^, while models 6 to 20 used four segmentations of FTIR wave number from 1000-1500 cm^-1 ^, 1500-2000 cm^-1^, 2000-2500 cm^-1^ and 2500-3000 cm^-1^. The data were subjected to ANN, LDA and PCA analysis.

**Table 1:** Optimized neuronal network parameters

Error goal	0.001
Transfer function of hidden layer	logsig
Number of hidden nodes	10
Training algorithm	Levenbery-Marqwardt
mu	0.001
Mu increase	10
Mu decrease	0.1

*Artificial neural network*

**Figure 4:**
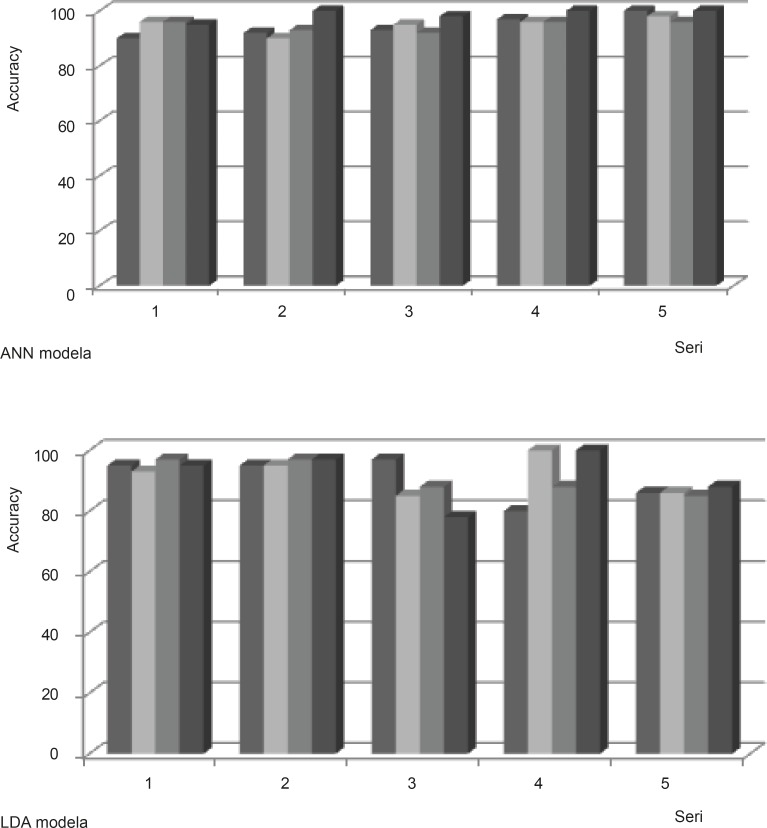
Distribution of predicted model with ANN and LDA in different series of dataset

We ran ANN on the dataset using Feed-forward backpropagation to analyze our networks. Training algorithms was obtained using Levenbery-Marqwardt back propagation algorithm. Three-layer neural networks was set, include one output layer, one hidden layer and an input layer. In order to determine the well optimized structure of the networks, error goal was selected 0.001% and verify number of hidden neurons were constructed. The parameters of the optimized neural network are listed in Table 1.When the model is performed for the training dataset in present investigation, Cell lines pattern of each experiment in the testing dataset is predicted in turn using the learned rules derived from the dataset in model training procedure. The 20 models were analyzed with ANN resulting in the classifications shown in Table 2. The results indicate that ANN is able to classify 90% of the resistance from sensitive cell lines, based on the FTIR data set. Comparison of the 20 ANN models indicates that the ANN using variables in segmentations of 1000-2000 cm^-1 ^fragment was more accurate than the other ANN models for the discrimination of sensitive versus resistant cells (Figure 4).

**Table 2:** Classification of FTIR data set of test (n =16; 8 A2780 and 8 A2780-CP) by Linear Discriminate Models and Artificial Neural

**Series**	**Model** **Train cell** **lines (** **n=44; 22 A2780 and 22 A2780-CP)**	**Artificial neural network**	**Linear discriminate analysis**
		**percent of correctly classified cell lines**	**percent of correctly classified cell lines**
**Seri 1**	Models trained with variables in 1000-3000 cm^-1^		
	1	90	95
	2	96	93
	3	96	97
	4	95	95
**Seri 2**	Models trained with variables in 3000-2500 cm^-1^		
	5	92	95
	6	90	95
	7	93	97
	8	100	97
**Seri 3**	Models trained with variables in 2500-2000 cm^-1^		
	9	93	97
	10	95	85
	11	92	88
	12	98	78
**Seri 4**	Models trained with variables in 1500-2000 cm^-1^		
	13	97	80
	13	96	100
	13	96	88
	16	100	100
**Seri 5**	Models trained with variables in 1000-1500 cm^-1^		
	17	100	86
	18	98	86
	19	96	85
	20	100	88

*LDA analysis*

LDA was also used to analyze the same 20 data sets of FTIR spectra values. The results of these analyses are given in Table 2. Classification rates provided by the LDA models were about 85%. Comparison of the 20 LDA models indicates that using variables in segmentations of 2500-3000 cm^-1 ^fragment was more accurate and less variable than the other LDA models (Figure 4). This might represent that the CH stretching region (3000-2500 cm^-1^) contains the asymmetric and symmetric membrane lipids (28) have linear pattern in resistant and sensitive cell lines.

*Comparison of LDA and ANN*

The comparison between LDA and ANN were done using paired student t-test. From the result of the t-test, it is obvious that the prediction accuracy in ANN models are different from the accuracy of LDA models with p-value ≤ 0.02. The data set between 1000-2000 cm^-1^ is more correctly classified with ANN model while the data set between 2500-3000 cm^-1^ is a better candidate for LDA model. According to total data sets used, the ANN modeling performs better than LDA because of less variation. Our analyses demonstrate that it is possible to classify individual resistant cell lines from sensitive type based on the analysis FTIR spectra using multivariate ANN analysis.

**Figure 5:**
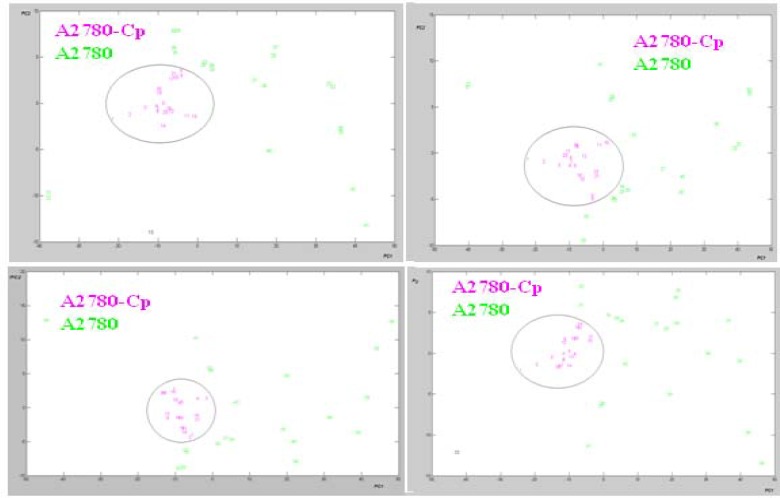
Score plot of PCA analysis in the four region of data sets resulted from FTIR spectroscopy of cisplatin sensitive A2780 and resistant A2780CP cell lines

*PCA analysis*

PCA can be used to extract the most significant variations between groups of spectra of cells. Score plots in PCA model provide visualization of the data, whereby the loading of data is an indicator of biochemical similarity (29). PCA was used to analyze the same 20 data sets. There are no suitable clustering with PCA for Seri 2 to 5 of data set (data was not shown). PCA was used to analyze the total data sets (Seri 1) extracted from FTIR spectra values. The cluster of points derived from the first two PC scores which summarized spectral features of two cell lines are shown in a 2-dimensional projection (Figure 5).

**Figure 6:**
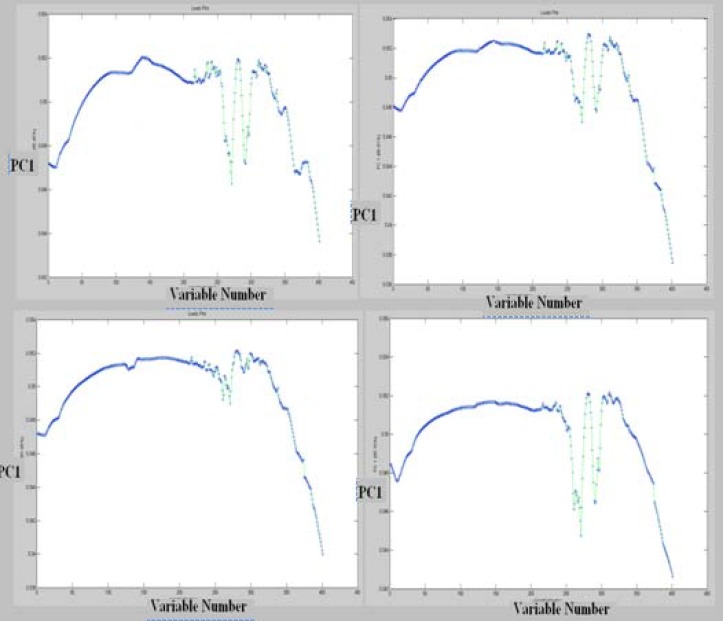
Loading plot of PCA analysis in the four region of data sets resulted from FTIR spectroscopy of cisplatin sensitive A2780 and resistant A2780CP cell lines

The data of the resistant cells are in the central area of PCA projection. Based on this approach, the PCA correctly classified more than 95% of all spectra for representing the variety of cell line spectra. Thus PCA as unsupervised model provide a good separation for representing the variety of sensitive and resistant cell line spectrum between 1000-3000 cm^-1^. Moreover Figure 6 shows the loading plot of PC1 from four fragmented observation in these cell lines.

**Figure 7:**
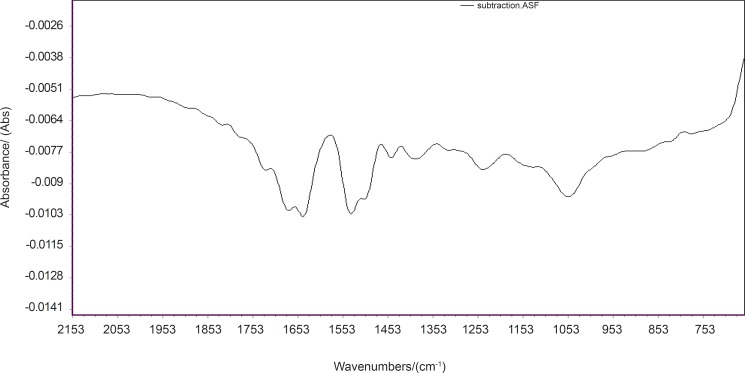
Biochemical typicality spectra of A2780 and A2780-CP cell lines

Analysis through direct observation of the spectra is not an easy task. Biochemical discriminatory spectra were calculated for the difference between the spectra of resistant and sensitive cells (Figure 7). Based on this result, most variation are in the band of 1580 cm^-1 ^could be related to amid II (23).The pattern of biochemical discriminatory spectra is found to be the same as loading plot. Our analyses demonstrate that loading of PCA model is a good approach to show FTIR discriminatory patterns of spectra markers.

## Conclusions

Resistance to Chemotherapy is a serious obstacle in treatment of cancer. In ovarian cancers, greater than 70% of patients initially respond to therapy with Cisplatin. However, this stabilized the five-year survival rate for ovarian carcinoma population less than 25% (25). This study suggests that infrared spectroscopy and analyzing data with classificatory methods may represent a biochemical pattern of resistant in cell lines. Identifying biochemical pattern of resistant cells may bring new tool to follow sensitivity of cancer cells in the duration of treatment so that a higher concentration of drug, and/or other effective chemotherapeutic agents to be used in saving time and better therapeutic outcome.

Cluster separation of FTIR spectrum data in PCA, ANN and LDA was found in previous studies. Discrimination rates have been reported verify between 60 to 98% of data with multivariate analysis of FTIR data (29, 30). This study suggests that infrared spectra in sensitive and resistant cell with PCA, LDA and ANN model discriminate more than 90% of data. In supervised model it is obvious that LDA models were variable and less accurate than those provided by ANN. LDA models appear to handle the classification problems in the fragment of 2500-3000 cm^-1^ with less variability which addresses linear alteration in the CH stretching region of resistant and sensitive cells. We offer PCA among unsupervised modeling and ANN as supervised modeling for pattern recognition of sensitive and resistant type.

## References

[1] Notani PN (2001). Global variation in cancer incidence and mortality. Curr. Sci.

[2] Rabik CA, Dolan ME (2007). Molecular mechanisms of resistance and toxicity associated with platinating agents. Cancer Treat. Rev.

[3] Paston I, Gottesman M (1987). Multiple-drug resistance in human cancer. N. Engl. J. Med.

[4] Leung EL, Fraser M, Fiscus RR, Tsang BK (2008). Cisplatin alter nitric oxide synthesis levels in human ovarian cancer cells: involvement in p53 regulation and cisplatin resistance. Brit. J. Cancer.

[5] Tummala R, Diegelman P, Fiuza SM (2010). Characterization of Pt, Pd-spermine complexes for their effect on polyamine pathway and cisplatin resistance in A2780 ovarian carcinoma cells. Oncol. Rep.

[6] Manoharan R, Wang Y, Feld MS (1996). Histochemical analysis of biological tissues using Raman spectroscopy. Spectrochim. Acta (Part A) Mol. Biomol. Spectrosc.

[7] Wagnieres G, Star WM, Wilson B (1998). In-vivo fluorescence spectroscopy and imaging for oncological applications. Photochem. Photobiol.

[8] Mantsch HH, Choo-Smith LP, Shaw RA (2002). Vibrational spectroscopy and medicine: an alliance in the making. Vibr. Spectrosc.

[9] Ofir K, Kelly S, Yechiel S (2006). Functional and structural characterization of HIV-1 gp41 ectodomain regions in phospholipid membranes suggests that the fusion-active conformation is extended. J. Mol. Biol.

[10] Krafft C, Sobottka B, Geiger D (2007). Classification of malignant gliomas by infrared spectroscopic imaging and linear discriminant analysis. Anal. Bioanal. Chem.

[11] Steller W, Einenkel J, Lars-Christian H (2006). Delimitation of squamous cell cervical carcinoma using infrared microspectroscopic imaging. Anal. Bioanal. Chem.

[12] Johnson HE, Broadhurst D, Kell DB, Theodorou MK, Merry RJ, Griffith GW (2004). High-throughput metabolic fingerprinting of legume silage fermentations via Fourier transform. Infrared Spectroscopy and Chemometrics.

[13] Ellis DI, Broadhurst D, Kell DB, Rowland JJ, Goodacre R (2002). Rapid and quantitative detection of the microbial spoilage of meat by Fourier transform infrared spectroscopy and machine learning. Am. Soc. Microbiol.

[14] Marchevsky AM, Tsou JA, Laird-Offringa IA (2004). Classification of individual lung cancer cell lines based on DNA methylation markers. J. Mol. Diagnos.

[15] Marchevsky AM, Patel S, Wiley KJ, Stephenson MA, Gondo M, Brown RW, Yi ES, Benedict WF, Anton RC, Cagle PT (1998). Artificial neural networks and logistic regression as tools for prediction of survival in patients with stages I , II non-small cell lung cancer. Mod. Pathol.

[16] Cenci M, Nagar C, Vecchione A (2000). PAPNET-assisted primary screening of conventional cervical smears. Anticancer Res.

[17] Troni GM, Cipparrone I, Cariaggi MP, Ciatto S, Miccinesi G, Zappa M, Confortini M (2000). Detection of false-negative Pap smears using the PAPNET system. Tumori.

[18] Jahandideh S, Abdolmaleki P (2010). Prediction of melatonin excretion patterns in the rat exposed to ELF magnetic elds based on support vector machine and linear discriminant analysis. Micron.

[19] Farid EA (2005). Artificial neural networks for diagnosis and survival prediction in colon cancer. Molecular Cancer.

[20] Guo-Zheng L, Hua-Long B, Mary QY, Xue-Qiang Z (2008). Selecting subsets of newly extracted features from PCA and PLS in microarray data analysis. BMC Genomics.

[21] Safaak H (2009). Spectroscopic study for detection and grading of breast carcinoma in-vitro. Austral. J. Basic Appl. Sci.

[22] Baker MJ, Gazi E, Brown MD, Shanks JH, Gardner P, Clarke NW (2008). FTIR-based spectroscopic analysis in the identification of clinically aggressive prostate cancer. Brit. J. Cancer.

[23] Zendehdel R, Masoudi-Nejad A, Mohammadzadeh J, Shirazi FH (2012). Cisplatin resistant patterns in ovarian cell line using FTIR and principle component analysis. Iranian J. Pharm. Res.

[24] Tewari KS, Monk BJ (2005). Gynecologic oncology group trials of chemotherapy for metastatic and recurrent cervical cancer. Curr. Oncol. Rep.

[25] Mishra NN, Yang S, Sawa A, Rubio A (2009). Analysis of cell membrane characteristics of in-vitro-selected daptomycin-resistant strains of methicillin-resistant Staphylococcus aureus. Antimicrob. Agents Chemother.

[26] Kartalou M, Essigmann JM (2001). Mechanisms of resistance to cisplatin. Mutat.Res.

[27] Bosch A (2008). Fourier transforms infrared spectroscopy for rapid identification of nonfermenting Gram-negative bacteria isolated from sputum samples from cystic fibrosis patients. J.Clin. Microbiol.

[28] Zanier K, Ruhlmann C, Melin F, Masson M (2010). E6 proteins from diverse papillomaviruses self-associate both in-vitro and in-vivo. J. Mol. Biol.

[29] McCann MC, Defernez M, Urbanowicz BR (2007). Neural network analyses of infrared spectra for classifying cell wall architectures. Plant Physiol.

[30] Rebuffo-Scheer CA, Schmitt J, Scherer S (2007). Differentiation of network analysis of Fourier-transformed. Infrared Spectra Appl. Environ. Microbiol.

[31] Tangri N, Ansell D, Naimark D (2008). Predicting technique survival in peritoneal dialysis patients: comparing artificial neural networks and logistic regression. Nephrol. Dial. Transplant.

